# A non-invasive tool to collect small intestine content in post weaning pigs: validation study

**DOI:** 10.1038/s41598-024-59950-3

**Published:** 2024-04-30

**Authors:** Inés García Viñado, Federico Correa, Paolo Trevisi, Giuseppe Bee, Catherine Ollagnier

**Affiliations:** 1 Pig Research Unit, Agroscope, 1725 Posieux, Switzerland; 2 Department of Agricultural and Food Sciences (DISTAL), University of Bologna, 40127 Bologna, Italy

**Keywords:** Microbial communities, Gastrointestinal system

## Abstract

The Capsule for Sampling (CapSa) is an ingestible capsule that collects small intestine content while transiting through the natural digestive pathway. In this study, 14 Swiss Large White pigs weighing less than 12 kg (Category < 12 kg) and 12 weighing between 12 and 20 kg (Category [12–20 kg]) were given two CapSas and monitored for three days. The animals were euthanized for post-mortem sampling, allowing us to directly obtain gut microbiota samples from the gastrointestinal tract. This post-mortem approach enabled a direct comparison between the microbial content from the gut and the samples collected via the CapSas, and it also facilitated precise identification of the CapSas’ sampling sites within the gastrointestinal tract. For the category under 12 kg, only 2.3% of the administered CapSas were recovered from the feces. In contrast, in the 12–20 kg category, 62.5% of the CapSas were successfully retrieved from the feces within 48 h. Of these recovered CapSas, 73.3%—equating to 11 capsules from eight pigs—had a pH > 5.5 and were therefore selected for microbiome analysis. Bacterial composition of the CapSas was compared with that of the three segments of the small intestine, the large intestine and feces of the corresponding pig. The results were tested using a PERMANOVA model (Adonis) including sample type as a factor, and then pairwise comparisons were made. The bacterial composition found in the CapSas differed from that of the large intestine and feces (*P* < 0.01), while it did not differ from the first segment of the small intestine (*P* > 0.10). This study provides evidence that the CapSa effectively samples the intestinal microbiota from the upper section of the small intestine in post-weaning pigs. Furthermore, it was found that the collection of CapSas could only be successfully achieved in pigs classified within the heavier weight category.

## Introduction

The link between gut microbiota and pig health is well-documented^1,2^, with gut microorganisms playing crucial roles in immunity and nutrient digestion^3,4^. While existing studies predominantly examine fecal microbiota^5^, these do not accurately reflect the microbiota in other digestive tract segments like the small intestine^6,7^, where the composition varies by location and pig age^8,9^. This variation is significant, especially in the small intestine, for nutrient digestion and immune system development^10^. Understanding host-microbiota interactions requires studying the gut microbiota's spatio-temporal changes, emphasizing the need for precise, repeatable sampling methods.

There are several methods for sampling gut microbiota. Post-mortem sampling gives access to all segments of the digestive tract, but can only be performed once on the same individual. Fecal sampling, on the other hand, is non-invasive and can be repeated several times on the same individual, but only represents the fecal microbiota^11^. Additionally, there are other sampling methods that are considered highly invasive like endoscopy, biopsies and cannulated animals. Endoscopy allows multiple sampling of intestinal contents and tissues (biopsies), but can only be performed under general anaesthesia. Finally, some studies have used cannulated animals for repeated sampling of the small intestine content^12,13^. For ethical and practical reasons, these last two methods are not always easily feasible. In 2020, Tang et al*.* highlighted the need for new sampling methods that would allow multiple, non-invasive samplings of intestinal contents^14^.

Recent advancements in ingestible medical devices designed to traverse the digestive tract have enabled the detailed sampling and examination of the human gut microbiota^15–17^. These devices are either an osmotic pill^15–17^ or an enteric-coated bladder^16,17^. Their sampling mechanism is activated when they reach the small intestine. Existing devices have been developed for humans and none have been successfully tested in pigs.

The aim of this study is to validate a new capsule prototype (CapSa) for non-invasive sampling of the intestinal microbiota in pigs.

## Results

### CapSa innocuity

All pigs remained healthy throughout the study. No macroscopic tissue damage was observed following euthanasia related to CapSa administration and/or passage. Administration of the CapSas had no impact on the fecal scores or the occurrence of diarrhea, with none of the pigs exhibiting a fecal score higher than 1. Moreover, all CapSas recovered on the day of euthanasia were found in the stomach.

### CapSa administration and recovery

**Figure 1:**
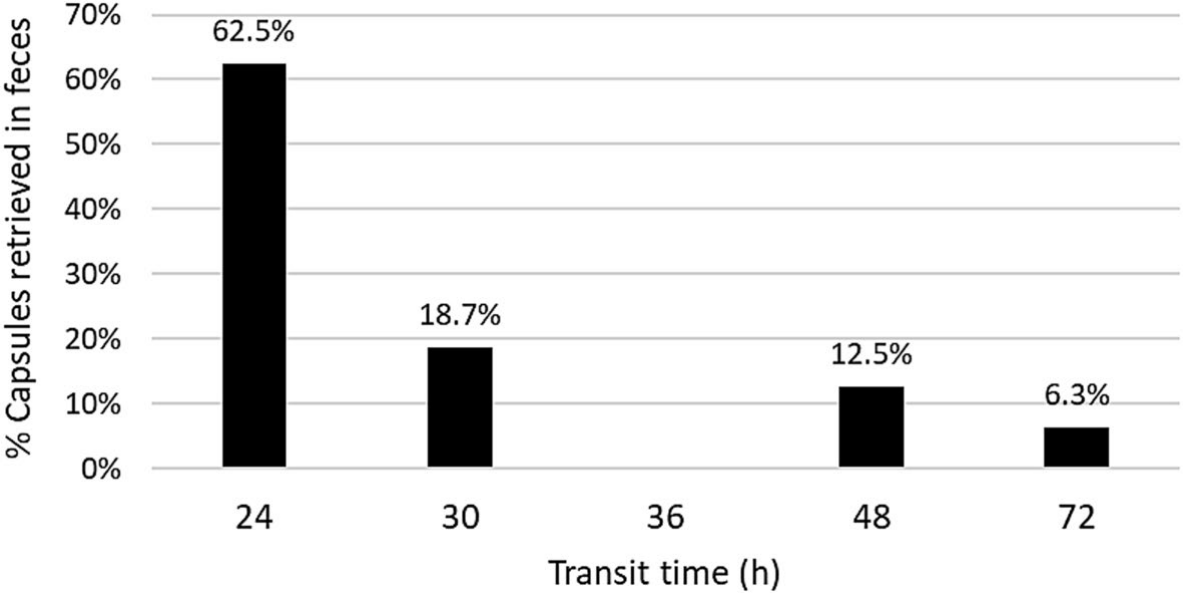
Time (h) of transit of CapSas found in feces. Time is calculated as the difference between the time of administration and the time of recovery.

Except for two pigs weighing less than 12 kg, pigs could successfully be administered with two CapSas. Weight category strongly influenced (*P* < 0.05) whether CapSa could be retrieved in the feces or in the digestive tract after slaughter. For pigs weighing less than 12 kg, 97.7% of the CapSas (24 capsules) were located in the stomach, 2.3% (1 capsule) were found in the feces (Table 1). In pigs weighing between 12 and 20 kg, 63.4% (15 capsules) were recovered from the feces, 17.6% (4 capsules) from the stomach, and 18.9% (5 capsules) were unaccounted for. In 62.5% of cases, CapSas found in feces transited within 24 h (Fig. 1). The percentage of CapSas not found was not affected by the weight category (*P* > 0.05). Sex had no impact on CapSa fate (*P* > 0.05).

**Table 1:** Outcomes of capsule administration by weight category.

	Weight category		SEM	*P*
	< 12kg	12–20kg		
Number of capsules found in feces	1	15	10.71	< 0.05
Number of capsules found in stomach	24	4	9.31	< 0.05
Number of capsules not found	0	5	13.60	0.34

SEM, Standard error of the mean; P, *P* value of the fixed effect.

### Characteristics of CapSas recovered from feces

**Figure 2:**
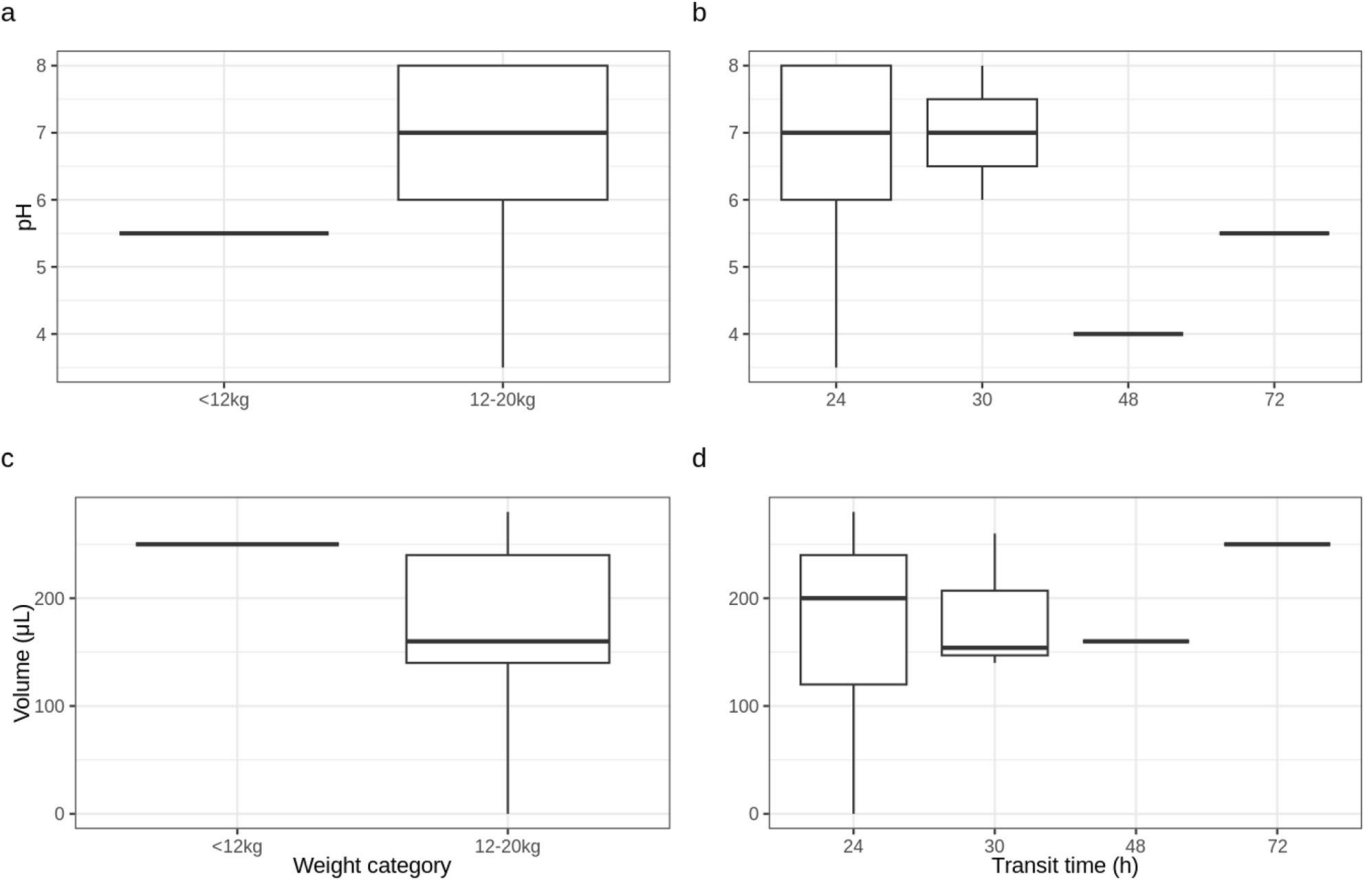
pH and volume (µL) of CapSa contents as a function of transit time (h) and pig weight category. Weight category had no effect (P = 0.14) on the pH of the contents of capsules found in feces (**a**), but transit time strongly influenced pH (P = 0.038), with pH falling below six when transit time exceeded 48 h (**b**). The volume samples collected was not affected by weight category (P = 0.44), sex (P = 0.11) or transit time (P = 0.59) (**c**, **d**).

Of the 16 CapSas recovered from the feces, two were broken and 12 (81.2%) had a pH > 5.5. The pH of the CapSa content was not affected (*P* > 0.05) by the body weight (BW) category (Fig. 2), but by the sex of the pigs (*P* = 0.048) and the transit time (*P* = 0.038). The pH of the CapSa content was < 6, when the transit time exceeded 48 h. The volume of digesta samples collected was not affected by the weight category, sex or transit time (*P* > 0.05). All CapSas recovered from feces within 48 h after administration and having a pH > 5.5 were considered for microbiota analysis.

### Microbiota analysis

A total of eleven CapSas collected from eight pigs weighing between 12 and 20 kg were sent for microbiota analysis. From these 11 samples, bacterial DNA was successfully extracted and amplified. Thereafter, DNA sequencing was performed for all but one CapSa that did not contain sufficient DNA.

**Figure 3:**
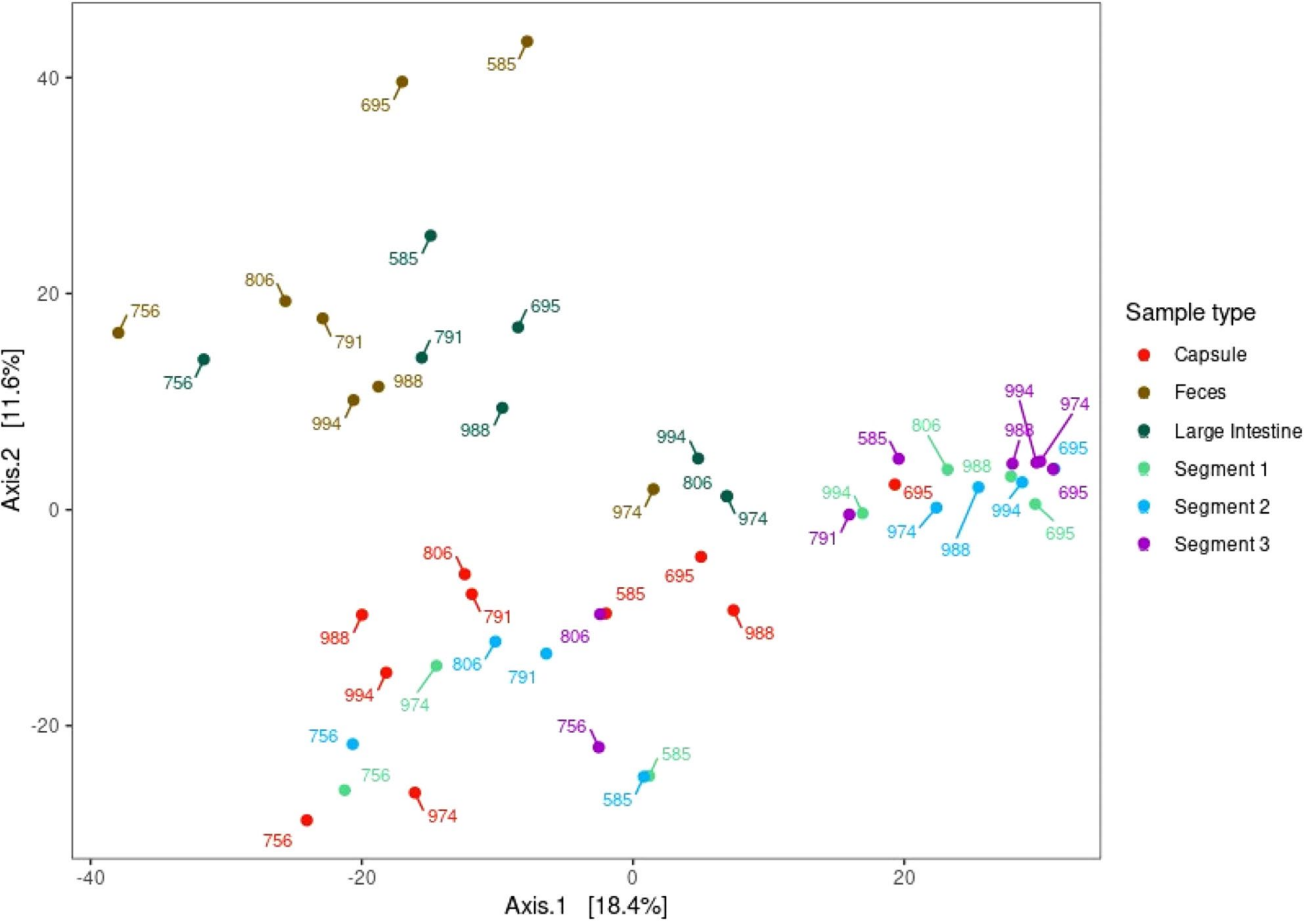
PCoA of Euclidean distance matrices of clr-transformed data. Samples are coloured based on sample type and labelled according to the subject.

Analysis of β-diversity by the Principal Coordinate Analysis (PCoA) demonstrated a clustering of microbiota according to sample type (Fig. 3). The large intestine and feces microbiota clustered together in a first cluster, while the microbiota from samples of the CapSas and the three segments (Segment 1, 2 and 3) of the small intestine tended to form a second cluster.

Based on the Adonis test, sample location and origin influenced the bacterial composition (*P* = 0.001). Pairwise comparisons detected differences in the bacterial composition between the CapSa contents, the contents of the three intestinal segments, the contents of the large intestine and feces (Table 2). According to this analysis, the bacterial composition of the three segments of the small intestine did not differ. There was also no difference between the bacterial composition of the large intestine and that of the feces. The microbial composition of the CapSa contents was different from that of the feces and large intestine, but not from that of Segment 1 (*P* = 0.32) and there was a tendency towards similarity with Segment 2 (*P* = 0.06) of the small intestine. It did, however, differ from that of Segment 3 (*P* = 0.01). A list of the ten main abundance Phylum, Family and Genera (expressed as % of total with the standard deviation) can be found as Supplementary Table S1.

**Table 2:** Comparisons of the bacterial compositions of the capsule contents, the three segments of the small intestine (Seg 1, 2 and 3), the large intestine and feces, using the Adonis test of Euclidean distances of clr-transformed data.

Pairwise comparisons	SumsOfSqs	F. Model	r^2^	*P*	*P* adj
Capsule vs Feces	7921.83	3.33	0.14	0.00	0.00
Capsule vs Large intestine	5007.93	2.56	0.11	0.00	0.00
Capsule vs Segment 3	6514.44	4.51	0.19	0.00	0.01
Capsule vs Segment 1	3623.75	2.06	0.10	0.02	0.32
Capsule vs Segment 2	4464.66	2.65	0.12	0.00	0.06
Feces vs Large intestine	3026.24	1.15	0.06	0.23	1.00
Feces vs Segment 3	9863.60	4.88	0.23	0.00	0.00
Feces vs Segment 1	8160.77	3.35	0.18	0.00	0.00
Feces vs Segment 2	8615.03	3.73	0.18	0.00	0.00
Large intestine vs Segment 3	4902.91	3.23	0.16	0.00	0.01
Large intestine vs Segment 1	3982.20	2.09	0.12	0.00	0.14
Large intestine vs Segment 2	4257.28	2.36	0.12	0.00	0.08
Segment 3 vs Segment 1	1280.95	1.02	0.06	0.32	1.00
Segment 3 vs Segment 2	1490.14	1.24	0.07	0.18	1.00
Segment 1 vs Segment 2	972.36	0.62	0.03	0.82	1.00

SumsOfSqs, Sum of square reflecting total variance; F. Model, F test value; r^2^, r-square value, reflects grouping differences, the higher the value, the higher the grouping differences; P, *P* value; P adj, *P* values adjusted for multiple comparison using the Bonferroni correction.

**Figure 4:**
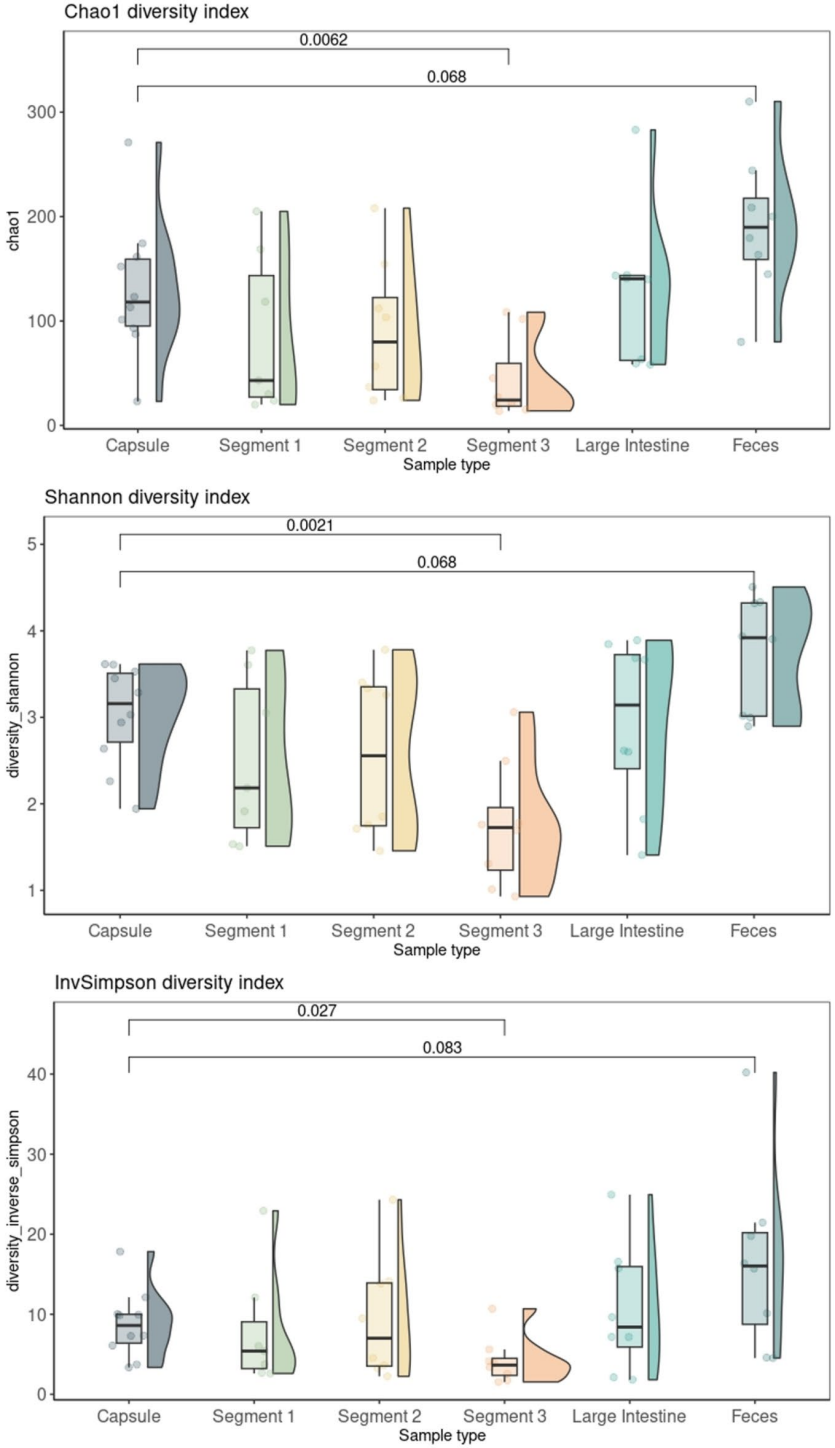
Box plots showing shows alpha diversity values for Chao1, Shannon and InvSimpson for each sample. Only *p* values < 0.10 are shown.

Figure 4 shows the alpha diversity values for Chao1, Shannon and InvSimpson indices for each sample. We compared the alpha diversity of the CapSa samples to that of other sample types. The overall bacterial richness (Chao1) was significantly higher in CapSa samples compared to Segment 3 (*P* < 0.10), tended to be lower compared to fecal samples (*P* = 0.06) and no differences were observed for all other comparisons. Similarly, Shannon diversity was higher in CapSa samples compared to Segment 3 (*P* < 0.01) and tended to be lower if compared with feces (*P* = 0.06), while no differences were observed for all other comparisons. The InvSimpson diversity was higher in CapSa compared to Segment 3 (*P* = 0.02) and tended to be lower if compared with feces (*P* = 0.08), while no differences were observed for all other comparisons.

## Discussion

This study validates the first non-invasive device to collect small intestinal content for microbiota analysis in post-weaning pigs. CapSas administered orally and then retrieved in the feces collected digesta with a pH > 5.5. As the pH of the fasting stomach does not exceed 5.5^18^, CapSa’s sampled the contents of a segment after the stomach. In addition, the in vitro studies showed that the majority of CapSas sample within one hour of being placed in an aqueous medium at pH = 7^19^. The in vivo sampling site is dependent on the increase of pH after passing through the stomach and its location depends on the speed of transit of the CapSa through the small intestine. Henze et al.^20^ reported that it took the SmartPill^®^ , an indigestible capsule of a slightly larger size (26 mm × 13 mm) 2.3 to 4 h to pass through the small intestine of male Landrace pigs weighing 15–17 kg. Together with the in vitro finding we conclude that CapSa sampling site is located in the small intestine.

The stomach appears to be the only site where CapSa can get trapped as no CapSas were found outside the stomach when examining the entire digestive tract *post-mortem*. In addition, prolonged transit time was associated with lower pH of the sampled content. We hypothesize that due to a prolonged retention time in the stomach, CapSa fail to resist acidic conditions which activates the sampling mechanism. Therefore, we conclude that content of CapSa with a pH < 5.5 could contain gastric content. Our findings align with the conclusions of the study by Rezaei Nejad et al.^15^, which found that capsules retrieved from the stomachs of pigs had a bacterial composition similar to that of the stomach content.

Expanding on this, we considered additional factors influencing the pH of the retrieved CapSa contents like the metabolization of the microbiome inside the capsule and the aggregation of SCFAs. Short-chain fatty acids, recognized as weak acids, are acknowledged contributors to pH modulation within the gastrointestinal tract. Notably, Hadinia et al.^21^ demonstrated a pH-dependent production of SCFA, with the highest and lowest amounts observed at pH 6 and pH 5, respectively. Another study by Henze et al.^22^ further underscored the influence of pH on SCFA production, particularly noting that a mildly acidic pH (5.5) stimulates specific SCFA production. Although this study did not directly assess fermentation within the CapSas, the existing literature suggests that the involvement of SCFAs in acidifying the CapSa contents cannot be ruled out.

Therefore, to ensure that CapSa contained digesta from the small intestine, only CapSas with a pH > 5.5 and a transit time of < 48 h were sent for DNA extraction and subsequent microbiota analysis. In our study, 11 CapSas from eight pigs met these requirements.

We then proceeded to validate the CapSa based microbiota composition. Applying the PCoA and the Adonis test, we demonstrated that the microbiota composition of the CapSa content is similar to that of Segment 1, tends to diverge from Segment 2, and is markedly distinct from that in Segment 3, large intestine and fecal samples. Previous studies have shown that the composition of microbiota varies significantly between different gastrointestinal segments and feces. Zhao et al*.*^6^ concluded that microbial profiles in feces were different from those in the small intestine. In addition, the microbial composition in the large intestine was more similar to feces than the small intestine, even across different pigs’ age^6^. Similarly, Adhikari et al*.*^23^ demonstrated the difference in microbiota composition between digesta samples from jejunum and colon on weaned piglets^6,23^. In the present study, the comparisons of the bacterial compositions of the three segments of the small intestine and feces showed a completely different composition. This further confirms that feces is not representative of the small intestinal microbiota.

Overall, it can be observed how bacterial richness and diversity tend to increase going from the small intestine to feces, these results are in line to what was observed in other studies^7,24^. In all three indices, the alpha diversity of the CapSa content did not differ from that observed in Segment 1 and 2 but was significantly higher than that in Segment 3. The similarity in alpha diversity between the CapSa content and Segment 1 indicates that the CapSa sample accurately mirrors the species diversity observed in Segment 1.

As the bacterial density in the lumen is higher than at or within the mucosa^25^, most mucosa-associated bacteria are represented in the luminal contents^26^ and many metabolites of interest are in the lumen. Due to its opening and closing mechanism and its movement via natural peristaltic motions, we hypothesized that CapSa samples luminal bacteria. These capsules therefore provide a non-invasive alternative to sample the content of the small intestine in pigs, which can be used for any laboratory analysis that can be performed with ~ 150µL.

To date, only a few sampling capsules were able to collect intestinal content. The sampling mechanism of the pill developed by Rezaei Nejad et al*.*^15^ was tested in weaned pigs and in macaques. They validated their sampling device in vivo by comparing the microbial composition profile of the capsule's sample to that of the matrix from which the capsule was retrieved. In their study, the bacterial composition of pills found in pigs stomach clustered with the stomach contents, while those found in feces clustered with the fecal microbial profile. The results in macaques were quite different, and the bacterial microbiome collected by the pill retrieved in feces was clearly different from feces. The authors concluded the pill was able to sample the regions of the gut upstream of the colon with quite distinct microbiome populations compared with the feces. Shalon et al*.*^17^ developed pill prototypes that can sample four different sites in human small intestine, from the duodenum to jejunum. To validate their device as a sampling tool to collect small intestine content, they attached one pill to a capsule endoscope and visualized successful video sampling in one human. They further confirm their results by observing differences between pills and saliva/stool samples, specifically in microbiota composition, prophage induction, protein abundance and bile acids profile.

With the aim of using CapSa for pigs, a standardised protocol was necessary to administer the capsules. Indeed, pig’s gastric emptying is very slow, and only small amounts of stomach content leave the stomach at once^27^. The speed of gastric emptying is highly variable between pigs, and large solid particles (> 1 cm) can remain in the stomach for several days^20^. The reason for this delayed emptying is anatomical. Indeed, pig’s stomach has a very pronounced “C” shape, and the gastric cardia is very close to the pylorus^27^. In addition, a transverse pyloric fold, called the *torus pyloricus*, is located right before the pylorus and serves as a “gate-keeper” to prevent any large particle to enter the small intestine^28^. To overcome the anatomical limitations, the administration protocol consisted in providing a liquid feed to limit gastrointestinal load, and a prokinetic to reinforce gastric contractions. A prokinetic is a substance that amplifies and coordinates the gastrointestinal muscular contractions to facilitate the transit^29^. Despite this protocol, pig’s BW still affected the percentage of CapSas found in the stomach, and consequently the percentage of CapSas recovered from feces. The smaller the piglet, the more likely the CapSa became stuck in the stomach. This explains why only pigs over 12 kg could be successfully sampled. As a consequence, CapSa is not an effective device in pigs with a bodyweight below 12kg since its retrieval in feces is impossible due to anatomical reasons.

The transit time did not distort bacterial composition. Indeed, CapSa content still had similar bacterial composition to Segment 1 of the small intestine, despite < 48h of transit under body temperature (38°C). Similarly, Shalon et al*.*^17^ demonstrated that there were no major changes in microbiota composition if transit (incubation) did not exceed 58 h.

This study validates the first non-invasive device for the collection and analysis of intestinal microbiota in post-weaning pigs with a bodyweight above 12 kg. A standardised protocol has also been established for successful deployment of the CapSas in pigs. This new tool opens new perspectives to study the gut physiology.

Further studies will be conducted to validate CapSa in fattening pigs as well as the effect of the protocol on the small intestine microbiota.

## Methods

### Animals and rearing conditions

For the study, 26 Swiss Large White pigs with BWs ranging from 6.4 to 20.0 kg were used. Fourteen weighed less than 12 kg (category < 12kg) and 12 weighed between 12 and 20 kg (category [12–20 kg]) (Table 3). Pigs of Category < 12kg were housed in groups of four and pigs of category [12–20kg] were housed in groups of two. All pigs had ad libitum access to a standard starter diet formulated to meet the nutritional requirements of post-weaning pigs^30^ (see Supplementary Table S2). Water was available ad libitum and distributed via nipple drinkers. The pens (total surface area of 4.47 m^2^) were specially designed to collect the CapSas by minimizing the slatted area and reducing the openings of the slatted area to a size smaller than the CapSa diameter.

**Table 3:** Characteristics of pigs included.

Weight category	N =	BW ± SD	%M	%F
< 12 kg	14	8.3 ± 1.6	50.0	50.0
[12–20 kg]	12	14.4 ± 2.6	58.3	41.7

. BW, Body Weight; SD, Standard Deviation; %M, Percentage of castrated males; %F, Percentage of females.

### Description of the capsule (CapSa)

The capsule studied is 21.7 mm long with a diameter of seven mm, corresponding to a size 0 hard capsule. CapSa opens, collects the sample and closes depending on the physico-chemical properties of its environment. It moves along the digestive tract purely passively, and the speed at which it passes depends entirely on intestinal peristalsis. CapSa can collect a maximum of 400 µL.

**Figure 5:**
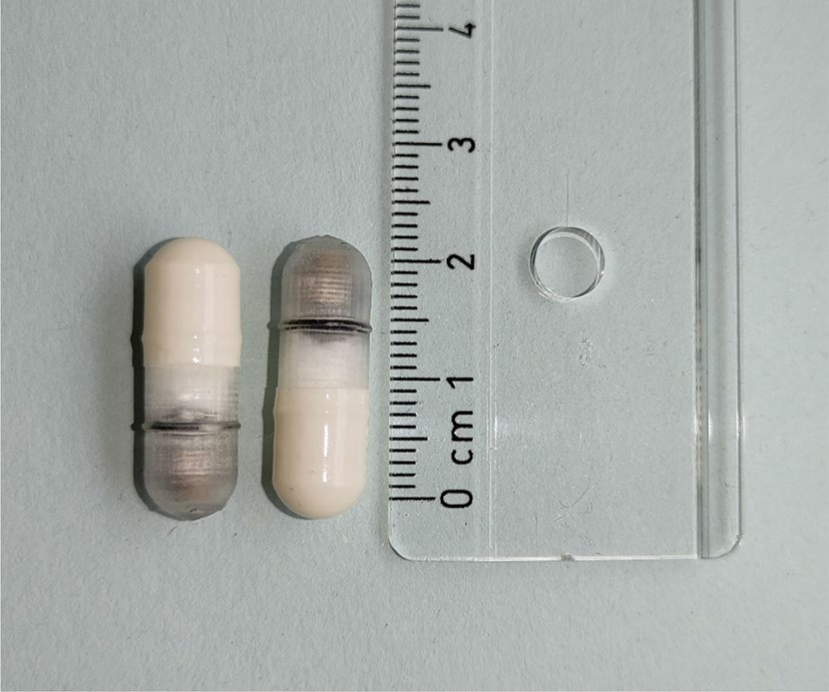
Capsule for Sampling (CapSa) device a rule is included for size comparison.

CapSa is designed to operate as follows: Once ingested, it moves through the stomach and reaches the small intestine, where it opens to collect a sample. After collection, the CapSa closes, continues its journey through the large intestine, and is finally expelled with the stool. This single-use device is specifically designed to collect fluid samples from the intestines for later ex vivo analysis. As illustrated in Fig. 5, the CapSa is composed of two main components: a dissolvable exterior with an enteric coating and a 3D printed bottom part. The CapSa is engineered to open when it encounters a pH level greater than 6, as the upper part dissolves, permitting intestinal fluids to enter. The entering intestinal content triggers the plunger to expand, which draws luminal content into the CapSa’s inner chamber. The CapSa is designed to automatically seal once the plunger mechanism is fully extended. In vitro results show that CapSa can withstand two hours in an acidic-aqueous medium (pH < 3), and then samples within an hour of being transferred to an aqueous medium at pH = 7^19^.

### Preparation of the animals and administration of CapSa

Two days prior to CapSa administration, three measures were taken to reduce intestinal load and shorten transit time. Firstly, two days before administering the CapSa (-2d), pigs were fed the starter diet in liquid form (ratio 1 kg of starter diet mixed with 2 L of water) and straw was removed from the pens. Secondly, one day before administering the CapSa (-1d) pigs had access to only half of their ration of feed and the feed was removed 12 h before capsules administration. Thirdly, to increase gastric emptying and thus facilitate CapSa transit through the stomach, 0.16 ± 0.015 mg/kg BW of prucalopride (Resolor^®^, Takeda Pharma AG, Glattpark, Switzerland) was administered orally via an oesophageal probe 40 min prior to administration. Prucalopride is a 5-HT_4_ serotonin agonist, which stimulates gastrointestinal peristalsis and increases gastric emptying^31–34^.

On the day of administration (0d), each pig received 2 CapSas. The capsules were administered by oesophageal sondage, while the pigs were kept in a sling adapted to their weight. A 10 mL bolus of orange juice was then administered to flush the capsule in the stomach. Every CapSa was assigned a unique number, linking it to the pig ID.

### CapSa recovery and sample processing

From 0d to three days after administration (3d), pens were inspected five times a day to look for CapSas expelled in the feces. The specifically designed slatted area of the pens allowed the capsule searches by sieving feces with water over the slatted surface. When CapSas were retrieved from the feces, they were directly transported to the laboratory. The outside was cleaned with 70% alcohol to avoid contamination after the opening. The identification of the CapSa was recorded and its content was extracted and the volume of the content determined. The content was transferred to a 0.5 mL Eppendorf (Eppendorf SE, Hamburg, Germany) immersed in liquid nitrogen and stored at -80°C until analysis. The pH of the content was measured using Litmus paper (Merck KGaA, Darmstadt, Germany) by cleaning the inside of the capsule after extraction.

### Post-mortem sampling

Three days after CapSa administration, all pigs were euthanised by electronarcosis. The gastrointestinal tract was extracted and starting right after the stomach the small intestine was divided into three segments (Segment 1, 2 and 3) of equal size. Samples of the three segments as well as the contents of the large intestine and feces were collected in sterile 2 mL Eppendorfs. The Eppendorfs were immersed in liquid nitrogen and stored at -80°C until analysis. The entire digestive tract was then inspected for capsules that had not yet been retrieved.

### Evaluation of CapSa innocuity

To assess the innocuity of CapSa administration, passage and retrieval, various parameters were considered to ensure the well-being of the pigs. Post-mortem macroscopic observations were conducted to assess the presence of any tissue damage related to CapSa administration and/or passage (e.g.; gastric ulcers, intestinal perforations, etc.). The fecal score was determined using a 4-level scoring scale, as follows: 1 = normal (firm but not hard); 2 = soft (does not hold form, piles but spreads slightly); 3 = runny (spreads readily); and 4 = watery (liquid consistency, splatters). Throughout the study duration, the overall health of the pigs was monitored continuously.

### Microbiota analysis

Only CapSa samples with a pH > 5.5 and recovered within 48 h of administration were used for microbiome analysis.

Bacterial DNA was extracted using the HostZERO™ Microbial DNA Kit (Zymo Research, California, USA) following the manufacturer's instructions. The DNA concentration (ng/µL) and purity (absorbance ratio 260/280 and 260/230, respectively) were verified spectrophotometrically on NanoDrop™ (Fisher Scientific, 13 Schwerte, Germany). The V3-V4 region of the 16S rRNA gene (~ 460 bp) was amplified by PCR using Platinum™ Taq DNA Polymerase High Fidelity (Termo Fisher Scientific, Italy) and the universal primers Pro341F: 5′-TCGTCGGCAGCGTCAGATGTGTATAAGAGACAGCCTACG GGNBGCASCAG-3′ and Pro805R:5′-GTCTCGTGGGCTCGGA GATGTGTATAAGAGACAGGACTACNVGGGTATCTAATCC-3′^35^. The PCR reaction conditions for amplification of DNA were as follows: initial denaturation at 94 °C for 1′, followed by 25 cycles of denaturation at 94 °C for 30”, annealing at 55 °C for 30” and extension 65 °C for 45”, ending with 1 cycle at 68°C for 7′^35^. Amplicons were then sequenced by Illumina MisSeq 300 × 2 bp with the MiSeq^®^ V3-V4 reagent kit on the MiSeq-Illumina^®^ platform. Microbiota analysis was performed using the DADA2 pipeline^36^ according to the Silva database taxonomy, version 138^37^. For the DADA2 pipeline, primers were removed from the raw sequences and based on the average quality score, forward and reverse reads were trimmed at position 280 and 260. All other DADA2 parameters were left with their default settings.

### Statistical analysis

All statistical analyses were performed in R (v 4.3.1). The percentage of CapSas recovered in feces, stomach or not found was calculated based on the total number of CapSas administered. Percentages were analysed using linear regression with BW category, sex and their interaction as fixed effects. An ANOVA was performed to check the effect of BW category and sex on the outcome of the CapSas. CapSa transit time was calculated as the time between capsule administration and recovery in the feces. CapSa pH and volume were analysed using linear regression with BW category, sex, transit time and the BW category x sex interaction as fixed effects. An ANOVA was performed to check the effect of BW category, sex and time of retrieval on the pH and volume of the sample of the retrieved CapSas. Interactions were removed if not significant at a *P* > 0.05.

Statistical analysis of alpha and beta diversity, as well as taxonomic analysis was performed using “phyloseq”^38^ v1.38, “vegan” v2.6^39^ and “microbiomeutilities” v1.0. For the alpha diversity samples were rarefied to the lowest sample depth, to avoid bias linked to different sampling efforts. Differences for alpha diversity indices (Chao1, Shannon, and Simpson diversity) between CapSa samples and other samples were assessed using the Wilcoxon test. For beta diversity, a dissimilarity matrix using Euclidean distances from the centred log-transformed (clr) data was constructed and the results represented using the PCoA plot. Differences were tested using a PERMANOVA model (Adonis) with 9999 permutations, including sample type as a factor. Pairwise contrasts between sample types were performed using the pairwise Adonis function included in the “PairwiseAdonis” package^40^. *P* values were then adjusted for multiple comparisons using the Bonferroni correction.

For all statistical analyses, a difference was declared significant if the *P* value < 0.05 and a trend was considered when 0.05 < *P* < 0.10.

### Ethics approval

All experimental procedures were in compliance with Swiss animal welfare guidelines and were approved (No. 2021-39-FR) by the Cantonal Veterinary Office of Fribourg (Switzerland). All methods are reported in accordance with the ARRIVE guidelines.

### Supplementary Information

Supplementary Information 1.

Supplementary Information 2.

## Data Availability

The datasets generated during and/or analysed during the current study are available from the corresponding author on reasonable request. Raw sequences are available at NCBI sequence read archive (SRA under the accession number PRJNA1049758).
