# Supplementary material for: A non-invasive tool to collect small intestine content in post weaning pigs: validation study
Source: Sci Rep. 2024 Apr 30;14:9964. doi: 10.1038/s41598-024-59950-3 (PMC11063154; doi:10.1038/s41598-024-59950-3)
Supplement: Supplementary file 1 — Supplementary Information 1. [file 41598_2024_59950_MOESM1_ESM.docx]

# Supplementary Table S1

# Relative abundance (expressed as % of total) and standard deviation of the main Phyla, Family and Genera present in the different microbiota samples

|  | **Sample type** | | | | | |
| --- | --- | --- | --- | --- | --- | --- |
|  | **Capsule** | **Segment 1** | **Segment 2** | **Segment 3** | **Large intestine** | **Feces** |
| **Main Phyla (%) ± SD^1^** |  |  |  |  |  |  |
| Firmicutes | 93.25 ± 6.10 | 96.65 ± 6.1 | 97.57 ± 2.61 | 98.87 ± 2.36 | 97.33 ± 2.73 | 92.27 ± 6.41 |
| Euryarchaeota | 3.88 ± 3.92 | 1.00 ± 3.92 | 0.83 ± 1.18 | 0.30 ± 0.72 | 0.26 ± 0.28 | 1.37 ± 1.07 |
| Proteobacteria | 1.32 ± 4.27 | 0.43 ± 4.27 | 0.17 ± 0.39 | 0.02 ± 0.03 | 0.43 ±0.79 | 0.12 ± 0.15 |
| Actinobacteriota | 1.02 ± 1.28 | 1.68 ± 1.28 | 1.17 ± 1.85 | 0.72 ± 2.23 | 0.07 ± 0.10 | 0.11 ± 0.17 |
| Bacteroidota | 0.40 ± 1.34 | 0.07 ± 0.16 | 0.03 ± 0.04 | 0.02 ± 0.02 | 1.50 ± 2.08 | 4.98 ± 4.31 |
| Patescibacteria | 0.07 ± 0.23 | 0.05 ± 0.11 | 0.06 ± 0.14 | 0.02 ± 0.09 | 0.07 ± 0.13 | 0.12 ± 0.14 |
| Cyanobacteria | 0.04 ± 0.05 | 0.10 ± 0.19 | 0.08 ± 0.16 | 0.00 ± 0.00 | 0.00 ± 0.00 | 0.05 ± 0.13 |
| Desulfobacterota | 0.01 ± 0.02 | 0.00 ± 0.00 | 0.00 ± 0.00 | 0.00 ± 0.02 | 0.06 ± 0.17 | 0.11 ± 0.12 |
| Spirochaetota | 0.00 ± 0.01 | 0.00 ± 0.01 | 0.00 ± 0.00 | 0.00 ± 0.00 | 0.07 ± 0.11 | 0.77 ± 2.13 |
| Campylobacterota | 0.00 ± 0.01 | 0.02 ± 0.08 | 0.09 ± 0.47 | 0.05 ± 0.24 | 0.19 ± 0.45 | 0.07 ± 0.11 |
| **Main Family (%) ± SD^1^** |  |  |  |  |  |  |
| Lactobacillaceae | 30.48 ± 18.17 | 76.63 ± 25.98 | 79.03 ± 23.39 | 73.87 ± 34.39 | 63.02 ± 22.45 | 47.79 ± 5.59 |
| Clostridiaceae | 12.72 ± 10.76 | 3.58 ± 5.46 | 1.74 ± 3.02 | 10.41 ± 18.79 | 3.10 ± 7.45 | 5.09 ± 7.33 |
| Lachnospiraceae | 11.24 ± 5.97 | 3.24 ± 5.34 | 5.38 ± 10.58 | 1.20 ± 2.95 | 11.63 ± 7.90 | 15.60 ± 8.85 |
| Peptostreptococcaceae | 11.09 ± 7.80 | 1.70 ± 1.07 | 0.86 ± 1.47 | 3.52 ± 8.11 | 2.51 ± 3.00 | 1.86 ± 2.03 |
| Streptococcaceae | 7.11 ± 7.73 | 5.77 ± 1.51 | 5.65 ± 10.42 | 6.50 ± 16.38 | 0.52 ± 1.06 | 0.68 ± 1.20 |
| Erysipelotrichaceae | 5.04 ± 4.12 | 0.71 ± 0.66 | 0.68 ± 1.01 | 1.60 ± 5.22 | 1.87 ± 2.11 | 1.92 ± 1.47 |
| Ruminococcaceae | 4.34 ± 3.22 | 1.11 ± 1.51 | 1.41 ± 2.76 | 0.40 ± 0.98 | 6.00 ± 4.07 | 6.28 ± 4.45 |
| Enterococcaceae | 4.27 ± 15.87 | 0.14 ± 0.66 | 0.00 ± 0.01 | 0.00 ± 0.01 | 0.00 ± 0.00 | 0.00 ± 0.00 |
| Methanobacteriaceae | 3.88 ± 3.92 | 1.00 ± 1.68 | 0.83 ± 1.18 | 0.30 ± 0.72 | 0.26 ± 0.28 | 1.37 ± 1.07 |
| Aerococcaceae | 2.19 ± 4.04 | 1.92 ± 4.07 | 1.13 ± 2.54 | 0.46 ± 1.52 | 0.00 ± 0.00 | 0.00 ± 0.00 |
| **Main Genera (%) ± SD^1^** |  |  |  |  |  |  |
| Lactobacillus | 19.63 ± 14.4 | 38.96 ± 23.69 | 27.33 ± 20.61 | 44.28 ± 26.7 | 51.81 ± 22.32 | 38.22 ± 21.98 |
| Clostridium sensu stricto 1 | 12.03 ± 9.99 | 1.81 ± 2.78 | 0.80 ± 1.45 | 9.60 ± 17.89 | 3.05 ± 7.06 | 5.00 ± 7.25 |
| Terrisporobacter | 9.41 ± 7.05 | 1.04 ± 1.69 | 0.65 ± 1.28 | 1.98 ± 5.00 | 2.04 ± 2.47 | 1.35 ± 1.41 |
| HT002 | 8.81 ± 6.65 | 28.26 ± 18.79 | 43.90 ± 25.17 | 25.48 ± 21.15 | 8.89 ± 5.92 | 8.50 ± 6.26 |
| Streptococcus | 7.11 ± 7.72 | 5.77 ± 12.41 | 5.65 ± 10.42 | 6.50 ± 16.38 | 0.52 ± 1.06 | 0.68 ± 1.20 |
| Blautia | 4.30 ± 2.42 | 1.58 ± 3.18 | 3.48 ± 7.82 | 0.67 ± 1.71 | 5.61 ± 6.30 | 5.30 ± 6.32 |
| Enterococcus | 4.27 ± 15.87 | 0.14 ±0.66 | 0.00 ± 0.01 | 0.00 ± 0.01 | 0.00 ± 0.00 | 0.00 ± 0.00 |
| Methanosphaera | 3.42 ± 3.89 | 0.89 ± 1.44 | 0.73 ± 1.02 | 0.27 ± 0.64 | 0.17 ± 0.20 | 0.96 ± 0.82 |
| Subdoligranulum | 2.81 ± 2.29 | 0.59 ± 1.03 | 1.00 ± 2.10 | 0.26 ± 0.63 | 3.34 ± 2.36 | 2.58 ± 2.35 |
| Turicibacter | 2.77 ± 3.16 | 0.21 ± 0.38 | 0.10 ± 0.33 | 1.46 ± 5.25 | 0.53 ± 1.16 | 0.23 ± 0.38 |

^1^ SD: Standard Deviation.
