# Supplementary material for: A non-invasive tool to collect small intestine content in post weaning pigs: validation study
Source: Sci Rep. 2024 Apr 30;14:9964. doi: 10.1038/s41598-024-59950-3 (PMC11063154; doi:10.1038/s41598-024-59950-3)
Supplement: Supplementary file 2 — Supplementary Information 2. [file 41598_2024_59950_MOESM2_ESM.docx]

# Supplementary Table S2

Ingredients and gross chemical composition of the post-weaning, starter.

|  | Post-weaning diet ^1^ |
| --- | --- |
| Ingredients (%) |  |
| Barley, ground | 6,426 |
| Oats, ground | 10,000 |
| Oats flakes | 2,000 |
| Maize | 3,442 |
| Wheat, kernels | 45,096 |
| Wheat starch | 0,688 |
| Cerolac 15-2175 | 5,000 |
| Rapeseed oil | 2,500 |
| Potato protein | 8,459 |
| Soya extract | 2,011 |
| Wheat bran | 0,506 |
| Beet pulp | 6,585 |
| Apple pomace, dried | 4,000 |
| L-lysine HCl | 0,401 |
| DL-methionine | 0,013 |
| L-threonine | 0,086 |
| Monocalcium phosphate | 0,476 |
| Salt | 0,390 |
| Ca-formate | 1,000 |
| Pellan^2^ | 0,300 |
| ALP-S 463 Piglets | 0,400 |
| Luctarom 3 | 0,010 |
| Greencab-70-C ^4^ | 0,200 |
| Natuphos 5000 G ^5^ | 0,010 |

Diet for the piglets from 15 days after birth to 14 days post-weaning, formulated according to the Swiss feeding recommendations for pig.

^2^ Pellet binding aid: Pellan, Mikro-Technik, Bürgstadt, Germany.

3 Luctarom, Lucta; Montornès del Vallès, Spain.

^4^ Coated calcium butyrate: Greencab 70-c, Brenntag; Denmark.

^5^ Phytase; 500 units of aspergillus niger phytase/kg diet; 1 phytase unit corresponds to the amount of enzyme that releases 1 μmol P from 5 mM phytate/min at pH 5.5 and 37˚C.

|  | Post-weaning diet ^1^ |
| --- | --- |
| Gross chemical composition analysed (g/kg as fed) |  |
| Dry matter | 887 |
| Crude protein | 170 |
| Fat | 45 |
| Crude fibre | 44 |
| Digestible energy (MJ/kg) | 14 |
| Lysine | 11 |
| Methionine | 3.1 |
| Threonine | 7.5 |
| Tryptophan | 1.9 |
| Ca | 5.8 |
| P | 4.5 |
| Na | 1.9 |
| Vitamin A (IE/kg) | 8000 |
| Vitamin D3 (IE/kg) | 1000 |
| Vitamin E | 25 |
| I (mg/kg) | 0.15 |
| Mn (mg/kg) | 10 |
| Cu (mg/kg) | 6 |
| Zn (mg/kg) | 75 |
| Se (mg/kg) | 0.20 |
